# Comment on the use of artificial intelligence in writing scientific papers

**DOI:** 10.1093/braincomms/fcad354

**Published:** 2023-12-23

**Authors:** Hinpetch Daungsupawong, Viroj Wiwanitkit

**Affiliations:** Private Academic Consultant, Lak 52. Phonhong, Vientiane 1000, Laos, Lao People's Democratic Republic; Department of Research Analytics, Saveetha Dental College and Hospitals, Saveetha Institute of Medical and Technical Sciences Saveetha University, Chennai 600077, India

We would like to comment on the Editorial ‘To ChatGPT or not to ChatGPT: the use of artificial intelligence in writing scientific papers.’^1^ The rise of artificial intelligence (AI) in academic paper creation is discussed in this article, with a particular focus on the AI chatbot ChatGPT. The shortcomings of ChatGPT, such as its construction approach and lack of transparency about information sources, are emphasized. The Editorial also expresses worry over the use of ChatGPT as a co-author in scientific articles, which violates standard authorship guidelines. Academic journal editors, publishers and preprint server administrators are realizing the importance of revising author guidelines to accommodate the usage of AI tools in article creation. Furthermore, the paper points out that using chatbots to generate text often results in low-quality output. Overall, it is not advised to use chatbots such as ChatGPT to produce content for academic papers. It may be acceptable that ChatGPT can cause a number of unanticipated problems. The most prevalent issues are the generated text's correctness and repeatability. Ethical issues may also exist, therefore it is vital to promote ethical AI use.^2^

The article might use more concrete examples and information to back up its statements concerning ChatGPT's limitations and low-quality outputs. It would also benefit from a discussion of potential alternatives or alternative approaches to utilize AI in academic writing. The article recommends that academic journal editors, publishers and preprint server administrators continue to revise their author requirements on this topic. Furthermore, more research and development is required to improve the quality and dependability of AI-generated content for academic purposes. According to the positive viewpoint, if ChatGPT is to be used in the future, good guidelines must be in place. Such standards must address both technological and ethical issues.

## Competing interests

The authors report no competing interests.

## Data Availability

Data sharing is not applicable to this article as no new data were created or analysed.
